# Non-Linear and Flexible Regions of the Human Notch1 Extracellular Domain Revealed by High-Resolution Structural Studies

**DOI:** 10.1016/j.str.2016.02.010

**Published:** 2016-04-05

**Authors:** Philip C. Weisshuhn, Devon Sheppard, Paul Taylor, Pat Whiteman, Susan M. Lea, Penny A. Handford, Christina Redfield

**Affiliations:** 1 Department of Biochemistry, University of Oxford, South Parks Road, Oxford OX1 3QU, UK; 2 Sir William Dunn School of Pathology, University of Oxford, South Parks Road, Oxford OX1 3RE, UK

## Abstract

The Notch receptor is a key component of a core metazoan signaling pathway activated by Delta/Serrate/Lag-2 ligands expressed on an adjacent cell. This results in a short-range signal with profound effects on cell-fate determination, cell proliferation, and cell death. Key to understanding receptor function is structural knowledge of the large extracellular portion of Notch which contains multiple repeats of epidermal growth factor (EGF)-like domains. Here we investigate the EGF4-13 region of human Notch1 (hN1) using a multidisciplinary approach. Ca^2+^-binding measurements, X-ray crystallography, {^1^H}-^15^N heteronuclear nuclear Overhauser effects, and residual dipolar couplings support a non-linear organization for the EGF4-13 region with a rigid, bent conformation for EGF4-7 and a single flexible linkage between EGF9 and EGF10. These data allow us to construct an informed model for EGF10-13 which, in conjunction with comparative binding studies, demonstrates that EGF10 has an important role in determining Notch receptor sensitivity to Dll-4.

## Highlights

• The EGF4-13 region of human Notch1 contains both bent and flexible interfaces
• This changes the classical view of the receptor as a rigid linear structure
• Extra interaction sites of Notch with ligand may occur along its longitudinal axis
• Interfaces next to ligand-binding site differentially modulate ligand interactions

## Graphical Abstract

**Figure:**
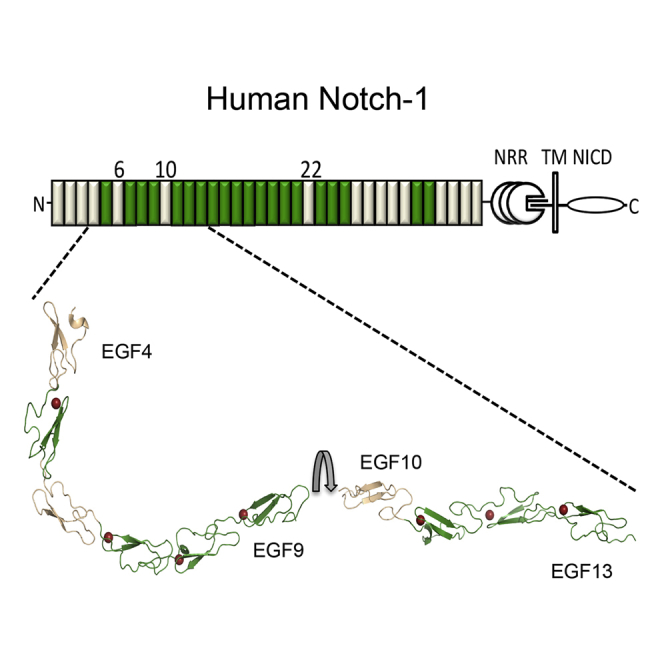

## Teaser

The structure of the extracellular domain of Notch receptor is key to understanding Notch biology. Weisshuhn et al. identify both bent and flexible regions within the receptor, changing the classical view of the receptor as a rigid linear structure extending from the cell surface.

## Introduction

The Notch pathway plays a key role in cell-fate determination, cell proliferation, and apoptosis during development with a crucial impact on most tissues and organs (Artavanis-Tsakonas et al., 1999, Bray, 2006). In adults, Notch has key roles in tissue homeostasis by regulating stem cell maintenance and function, immune system activation, and angiogenesis. The importance of the Notch pathway for human biology is underscored by the number of diseases resulting from its inappropriate activation or inhibition, including a number of inherited disorders and cancers (Louvi and Artavanis-Tsakonas, 2012, Ntziachristos et al., 2014).

**Figure 1:**
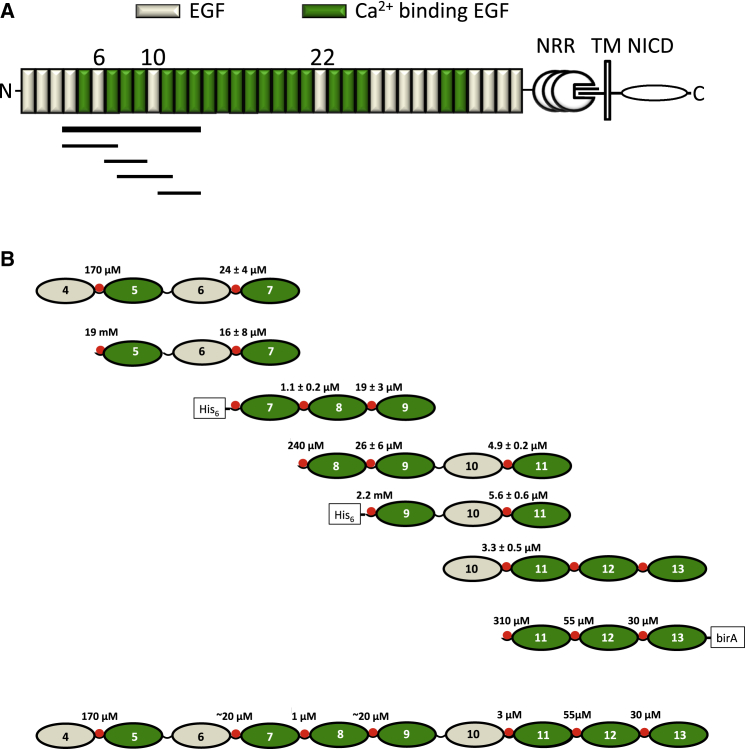
Modular Organization of the Extracellular Domain of Human Notch1 and Overview of Ca^2+^ Dissociation Constants (A) The negative regulatory region (NRR) and transmembrane domain (TM) of Notch1 are indicated. Individual domains belonging to the Notch intracellular domain (NICD) are not indicated separately. Ca^2+^-binding and non-Ca^2+^-binding EGF domains are indicated in green and wheat, respectively. The thick horizontal black line highlights the EGF4-13 region that is the subject of this study. The shorter lines indicate the principal constructs used here (EGF4-7, EGF7-9, EGF8-11, and EGF11-13). (B) The measured Ca^2+^ dissociation constants at pH 7.5 and *I* = 0.15 for all the constructs studied are shown. *K*_d_ values in the 1–20 μM range were determined by chromophoric chelation; at least three repeats were carried out and the experimental errors on the *K*_d_ values are shown. *K*_d_ values in the 20 μM to mM range were determined by NMR; repeat experiments were not carried out. Ca^2+^ is indicated by a red sphere at the N terminus of each Ca^2+^-binding EGF domain. EGF11-13 contains a recognition sequence for the site-specific biotinylation enzyme BirA at its C terminus. The N-terminal His_6_ tag has not been cleaved from EGF7-9 and EGF9-11. See also Figures S1 and S4.

Notch signaling requires cell-surface expression of a hetero-dimeric transmembrane Notch receptor, which has a large extracellular portion rich in epidermal growth factor (EGF)-like domains (36 in human Notch1 and *Drosophila* Notch) (Figure 1A). Ligand binding to Notch EGF11-12 by one of the two DSL ligand families (Jagged/Serrate or Delta) initiates regulated intramembrane proteolysis, where the receptor is cleaved within the negative regulatory region (NRR) by an ADAM metalloprotease and subsequently by the γ-secretase enzyme complex (Blaumueller et al., 1997, Gordon et al., 2007, Logeat et al., 1998, Sanchez-Irizarry et al., 2004). The final intramembrane cleavage releases the intracellular domain of Notch, which comprises RAM, ANK, and PEST sequences (Mumm and Kopan, 2000, Schroeter et al., 1998). This translocates to the nucleus, binds to a transcription factor of the CBF1, Suppressor of Hairless, Lag-1 (CSL) family, and, in the presence of co-activators such as Mastermind (MAM), relieves repression of genes of the HES and Hey families (Jarriault et al., 1995).

Interactions with the Notch receptor can activate or inhibit Notch signaling, dependent upon whether cell-surface ligands are presented to Notch on adjacent cells (in *trans*), or on the same cell (in *cis*) (deCelis and Bray, 1997, Franklin et al., 1999). Notch ligand activity is also sensitive to the modification of O-fucosylated Notch by Fringe (Moloney et al., 2000a, Moloney et al., 2000b, Rana and Haltiwanger, 2011, Shao et al., 2003). This can potentiate or prevent signaling by different ligands and this post-translational regulation is important in controlling embryonic patterning and boundary formation between adjacent developmental compartments (Johnston et al., 1997). In addition, O-glucosylation of Notch has been shown to be essential for activity (Acar et al., 2008).

Structural studies have informed on the ligand-binding EGF11-13 (Cordle et al., 2008a, Hambleton et al., 2004) and the NRR (Gordon et al., 2007) regions of the extracellular domain of Notch. Furthermore, the structure of the transcriptional complex formed by CSL, Notch intracellular domain (NICD), and MAM in combination with DNA has been solved (Nam et al., 2006, Wilson and Kovall, 2006); however, most of the extracellular region remains unsolved. New structural information for the EGF-rich regions of the receptor and, in particular, those flanking the ligand-binding region are essential to gain mechanistic insight into the processes of receptor activation and inhibition that occur when ligand is expressed in *trans* or *cis*, respectively, and to explain the effects of various mutations and post-translational modifications such as O-glycosylation. The three EGF domains of the ligand-binding site all adopt a canonical EGF fold and each contains a Ca^2+^-binding site at its N terminus, which together with a conserved hydrophobic packing interaction results in a near-linear and rigid conformation (Cordle et al., 2008a, Hambleton et al., 2004). Many of the other EGF domains are predicted to bind Ca^2+^ and, by homology to known structures, are expected to adopt extended inflexible structures similar to EGF11-13 (Downing et al., 1996, Handford et al., 1991, Rees et al., 1988). However, the multiple tandem repeats of Ca^2+^-binding EGF-like domains are interspersed with non-Ca^2+^-binding domains EGF6, EGF10, and EGF22, which may introduce sites of flexibility or adopt non-linear pairwise domain interactions (Figure 1A). An electron microscopy study suggested the existence of a Notch dimer with distinct conformational states (Kelly et al., 2010) but these data were obtained using affinity grid immobilization without conventional protein purification, and the Ca^2+^ concentration was ill defined. A “jack-knife” model for the receptor has been proposed to explain the genetic data (Xu et al., 2005), but there is as yet no direct experimental evidence for this, and a conformation that extends the receptor ectodomain away from the cell surface toward the ligand is also possible. Recently, the structure of EGF11-13 in complex with the N-terminal fragment of Dll-4 (NE1) has been reported, which shows the two molecules in an antiparallel orientation within the crystal (Luca et al., 2015). Furthermore, two distinct sites within EGF11-12 were shown to bind to Dll-4, with specific residues within EGF12 binding to the N-terminal C2 domain of the ligand and EGF11 residues making contacts with the DSL domain.

In this study, we have used nuclear magnetic resonance (NMR) spectroscopy and X-ray crystallography to investigate the structure and flexibility of the EGF4-13 region of the Notch ectodomain by analyzing a series of limited fragments with the non-Ca^2+^-binding EGF6 and EGF10 domains placed in a native context (Figure 1A). We report a crystal structure of EGF4-7, where the domain interface formed between the non-Ca^2+^-binding domain, EGF6, and its preceding domain introduces a bent conformation to the region. Residual dipolar coupling measurements are used to define interdomain orientations for other domain pairs in EGF4-13 and identify a single site of flexibility at the EGF9-10 linker. These data, together with Ca^2+^-binding and {^1^H} -^15^N heteronuclear nuclear Overhauser effect (NOE) measurements, allow modeling of EGF4-13, which suggests a non-linear, but not jack-knifed, organization. Superposition of EGF10-13 on EGF11-13 of the recently solved Notch/Dll-4 complex indicates that further interaction sites with ligand outside the core recognition site are possible, notably at EGF10/EGF1. Comparative binding analyses, by flow cytometry, indicate that the presence of EGF10 modulates the ability of the core recognition site to interact with Dll-4, but not Jagged1 (J1), revealing greater complexity to the molecular basis of ligand specificity than previously thought.

## Results

### Ca^2+^-Binding Measurements Reveal Rigid Interfaces for Ca^2+^-Binding EGF Domains in the EGF4-13 Region of Human Notch1

Ca^2+^ affinities for Ca^2+^-binding (cb) EGF domains of the EGF4-13 region of human Notch1 were measured to gain insight into the rigidity of interdomain interfaces in this region. In EGF domains, a consensus sequence of D/N-x-D/N-E/Q-x_m_-D/N^∗^-x_n_-Y/F (where ^∗^ indicates possible β-hydroxylation, and m/n are variable) is predictive for Ca^2+^ binding (Handford et al., 1991, Mayhew et al., 1992, Rand et al., 2000, Rees et al., 1988). Chromophoric chelation was used to measure *K*_d_ values for high-affinity sites (up to ∼20 μM (Jensen et al., 2005, Linse et al., 1991)), while NMR titrations were used to measure *K*_d_ values for low- and medium-affinity sites and to assign the high-affinity sites to specific EGF domains (Figure 1B) (Suk et al., 2004). Ca^2+^-binding EGF domains 5, 8, 11, 12, and 13 show the consensus Ca^2+^ binding sequence and the aromatic packing residue in the preceding domains. EGF domains 7 and 9 have an aspartic acid instead of the expected E/Q at the third consensus site (Figure S1).

Ca^2+^ affinities were measured in a number of constructs and the results are summarized in Figure 1B. N-terminal EGF domains have low affinity for Ca^2+^, and this is observed to increase when they are placed in a native context with a preceding EGF domain. For example, the affinity for Ca^2+^ of EGF5 is increased by ∼100-fold (from a *K*_d_ of 19 mM to a *K*_d_ of 170 μM) when it is preceded by EGF4. In the domains for which *K*_d_ values have been measured, only the presence of a preceding domain has an influence on the *K*_d_ value. For example, EGF9 has the same *K*_d_, within experimental error, in the EGF7-9 and EGF8-11 constructs. The *K*_d_ values for EGF domains 7, 8, 9, 11, 12, and 13 are in the range of ∼1–60 μM, and under the conditions of extracellular Ca^2+^ concentration (∼1.4 mM) (Breitwieser, 2008) these sites will be saturated to >∼95%. EGF5 has a weaker affinity for Ca^2+^ (*K*_d_ ∼170 μM); this site will still be occupied in ∼90% of molecules. The high Ca^2+^ affinity observed for all the cbEGF domains (including EGF7 and EGF9, which have aspartic acid instead of the expected E/Q at the third consensus site), and the observation that the affinity is enhanced by at least a factor of 50 when a preceding domain is present, suggests that the cbEGF domains studied here form a packing interaction with the preceding domain leading to a rigid interdomain interface.

### Crystal Structure of Human Notch1 EGF4-7 Reveals a Bent Conformation

**Figure 2:**
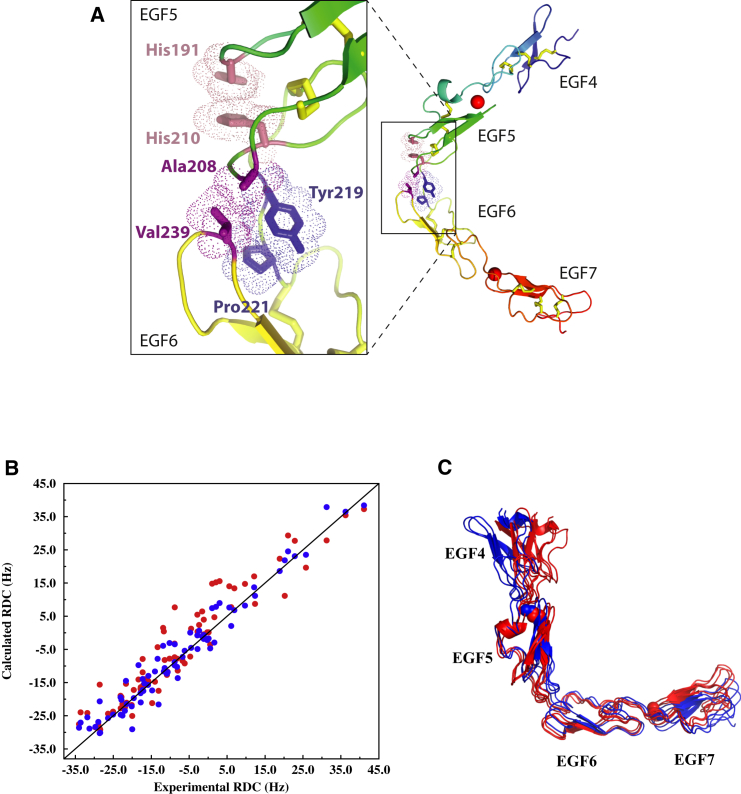
Structure of EGF4-7 Reveals the Bent Conformation of the EGF5-6 Junction (A) X-Ray structures of EGF4-7 (total of three independent chains in two crystal forms) reveal a consistent bent structure with the EGF5-6 junction adopting an ∼90° tilt angle. The main panel shows a representative structure (chain A from the P2_1_ crystal form) in a cartoon representation colored from blue at the N terminus to red at the C terminus. Ca^2+^ ions are shown as red spheres and the residues stabilizing the EGF5-6 junction highlighted in stick and van der Waals surface representations colored to highlight the side chains that pack together. The EGF5-6 junction is also shown in the zoom box with the same representation. (B) Comparison of experimental RDCs for EGF4-7 to RDC values calculated using the X-ray structure (P2_1_ A) (red circles) or a structure in which the EGF5-6 interdomain tilt angle was reduced from 90° to 70° (blue circles); the Q value decreases from 0.38 to 0.27 when the tilt angle is decreased. (C) Overlay of the three X-ray structures (red) and RDC-modeled structures (blue) for EGF4-7; the two structures in blue represent the range of tilt angles obtained from the RDC data using Monte Carlo simulations with an experimental error of 2 Hz (see also Table S2). The Ca^2+^ ions bound to EGF5 and EGF7 are shown as spheres. See also Figure S2 and Tables S1 and S2.

Structures of two crystal forms (P2_1_ and C2) were determined for hN1 EGF4-7 using X-ray crystallography (Table 1). Each domain within the construct displayed a canonical EGF fold, with a single Ca^2+^ bound, as expected, to EGF5 and EGF7 (Handford et al., 1991, Mayhew et al., 1992, Rees et al., 1988). An unusual tilt angle of ∼80°–90° was observed at the domain interface of EGF5 and EGF6 for both crystal forms resulting in a bent conformation for this stretch of EGF domains (Table 2 and Figure 2A). The EGF5-6 domain interface is stabilized in this orientation by packing of the side chain of A208 (with some contribution of T209) in EGF5 with V239 from EGF6. A second packing interaction is also evident between Y219 and P221 at the N terminus of EGF6 (Figure 2A). These interactions are very different from the interdomain packing typically observed in cbEGF-cbEGF pairs, and EGF-cbEGF pairs, which involves a conserved aromatic residue located between the fifth and sixth cysteine in the N-terminal domain packing against residues on the major β hairpin of the C-terminal domain, resulting in a rod-shaped conformation (Cordle et al., 2008a, Downing et al., 1996, Hambleton et al., 2004, Smallridge et al., 2003). H210, which is located at the position of the conserved packing aromatic residue in EGF5, is instead involved in an intradomain interaction with H191 that helps to stabilize the loop containing A208. The EGF4-5 and EGF6-7 pairs adopt a more elongated conformation than observed for EGF5-EGF6, but these pairs are not as elongated as the previously determined structure for EGF11-13 (Table 2).

### Heteronuclear NOE Measurements Show that Interdomain Linkers Are Not Flexible on a Fast Timescale

**Figure 3:**
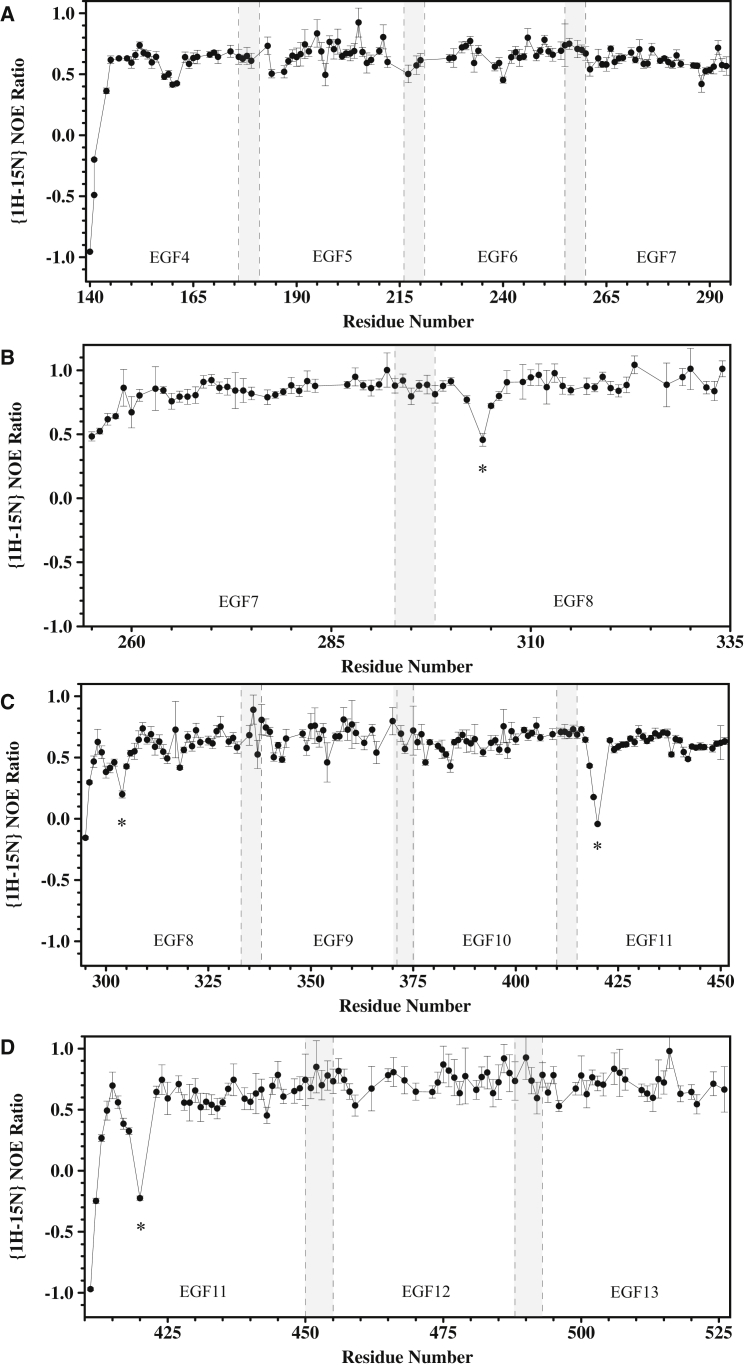
{^1^H} -^15^N Heteronuclear NOE Data for hN1 Constructs (A–D) Data are shown for (A) EGF4-7, (B) EGF7-9, (C) EGF8-11 and (D) EGF11-13. Reduced NOE ratios, characteristic of significant mobility on a nanosecond to picosecond timescale, are observed at the N terminus of each construct and for the loop between the first and second Cys in EGF8 and EGF11 (indicated by an asterisk). The regions highlighted by the dashed vertical lines and shading represent the linker between pairs of EGF domains (six residues between the sixth Cys of one domain and the first cysteine of the next for all linkers except EGF9-10 which has five residues). It is clear that reduced NOE ratios are not observed in any of these linkers, indicating that they are not flexible on a fast timescale. EGF4 shows reduced NOE values for residues 158–161. These residues are located in the β turn between the third and fourth Cys; in the X-ray structures of EGF4-7 these residues show high B factors or missing electron density suggesting dynamic behavior. Uncertainties in the NOE ratios were estimated from 500 Monte Carlo simulations using baseline noise as a measure of the error in the peak heights.

The {^1^H} -^15^N heteronuclear NOE provides a method for identifying regions of the polypeptide backbone that undergo fast timescale dynamics (picoseconds/nanoseconds) (Palmer, 2004). Data for the EGF4-7, EGF7-9, EGF8-11, and EGF11-13 constructs are shown in Figure 3. Reduced values of the heteronuclear NOE, characteristic of mobile residues, are observed for up to approximately four residues at the N terminus of each construct. EGF8 and EGF11 show reduced NOE values for some residues in the loop between the first and second cysteines. In both cases, this flexibility is observed when EGF8 or EGF11 is the N-terminal domain but also when it is preceded by EGF7 or EGF10. EGF8 and EGF11 have six residues in this loop in contrast to only four residues in Ca^2+^-binding EGF7, EGF9, EGF12, and EGF13 where flexibility is not observed. Other regions of the construct do not show evidence for fast timescale dynamics. In particular, the residues between the sixth cysteine of one domain and first cysteine of the following domain, which represent the interdomain linker, do not show evidence of low heteronuclear NOE ratios suggesting that interdomain flexibility, at least on a fast timescale (picosecond to nanosecond), is absent.

### Interdomain Orientations Determined using Residual Dipolar Couplings

Residual dipolar couplings (RDCs) are a useful NMR parameter for assessing the relative orientations of protein domains in solution and for identifying interdomain dynamics on a wider range of timescales than the heteronuclear NOE (Braddock et al., 2001, Chen and Tjandra, 2012, Fischer et al., 1999, Prestegard et al., 2004, Tolman and Ruan, 2006). ^1^H^N^-^15^N RDCs were measured for EGF4-7, EGF7-9, EGF8-11, and EGF11-13 using C12E6/*n*-hexanol as the alignment medium (Figure S2, and Tables S1 and S2) (Ruckert and Otting, 2000). The interdomain tilt and twist angles determined using these RDC data are summarized in Table 2.

#### Well-Defined Interfaces Observed for all Ca^2+^-Binding EGF Domains

The RDC data demonstrate that all Ca^2+^-binding EGF domains found in a native context have a well-defined and rigid interdomain interface; this is consistent with the conclusions from the Ca^2+^ affinity measurements. For the EGF4-5, EGF6-7, EGF11-12, and EGF12-13 pairs, the interdomain orientations in solution, as defined by the tilt and twist angles (Table 2), agree with the orientation observed in the X-ray structures of EGF4-7 and EGF11-13.

Crystal structures are not available for EGF7-9 and EGF8-11. The relative orientations of EGF8 and EGF9 with respect to EGF4-7 were determined from the RDCs measured for EGF7-9. The relative orientation of EGF10 with respect to EGF11-13 was determined from the RDCs measured for EGF8-11. The EGF7-8, EGF8-9, and EGF10-11 interfaces show the expected close proximity of the packing aromatic, found four residues after the fifth cysteine in the N-terminal domain, to the residues in the major β turn of the C-terminal domain (Figure S1); this packing interaction is expected in cbEGF domains with high affinity for Ca^2+^ as observed for EGF8, EGF9, and EGF11 (Figure 1B). The EGF7-8 pair has a tilt angle of 45°, showing a less-extended conformation than observed for EGF11-13. This domain pair has a non-standard packing interaction involving W287 in EGF7 and H316 in the β hairpin of EGF8, rather than the more common hydrophobic residue; this may influence the interdomain orientation. EGF10 adopts a less-extended conformation with respect to EGF11 than the remainder of the EGF11-13 construct with a tilt angle of 33° ± 10°.

#### EGF4-7 Is Bent in Solution

The X-ray structure of the EGF4-7 construct shows an unusual bent structure with a tilt angle of ∼80°–90° between EGF5 and EGF6 (Figure 2A). The RDC data for EGF4-7 suggest that the molecule tumbles in solution as a rigid object. In solution, the EGF5-6 interface is also observed to be bent but is somewhat more open (70° ± 2°) than the crystal structures (Figures 2B and 2C).

#### The EGF9-10 Interface Is Flexible

Attempts to fit the RDC data for the four domains of EGF8-11 to a single alignment tensor result in a significantly higher Q value than the individual fits of EGF8-9 and EGF10-11 (Figure S3). In addition, the D_a_ values, which define the alignment tensor, obtained from the fits of the RDCs for EGF8-9 and EGF10-11 are significantly different in both their magnitude and sign (D_a_ = 14.9 ± 0.5 for EGF8-9 and D_a_ = −8.5 ± 0.3 for EGF10-11) (Table S2). This suggests that the two pairs of domains align independently in solution in the EGF8-11 construct; such a result has previously been interpreted as indicating medium-to-large-scale interdomain motion (Braddock et al., 2001). Thus the EGF9-10 interface appears to be flexible, which is in line with the prediction of EGF10 as a non-Ca^2+^-binding domain (Hambleton et al., 2004), the absence of an aromatic consensus residue at position four after the fifth cysteine in EGF9, and the prediction by TALOS+ (Shen et al., 2009), on the basis of ^1^H, ^13^C and ^15^N chemical shifts, of lower order parameters for the residues at the beginning of EGF10. The absence of reduced heteronuclear NOE values for the EGF9-10 interface suggests that mobility of this interface is on a slower microsecond to millisecond timescale. Therefore, within the EGF4-13 region of human Notch1, the EGF9-10 interface is the only site of significant interdomain flexibility.

### Model of EGF4-13 Region of Human Notch1

**Figure 4:**
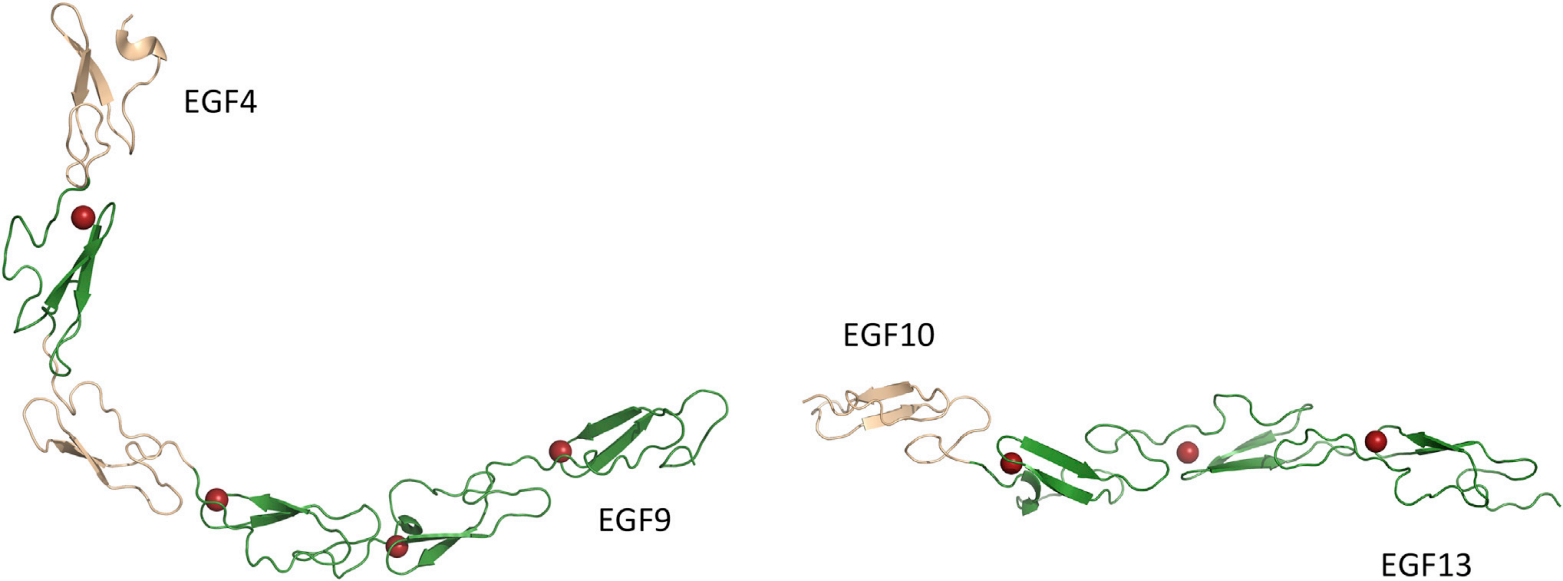
Models for the EGF4-13 Region of Human Notch1 The models for the EGF4-9 (left) and EGF10-13 (right) regions of human Notch1 are based on the X-ray structures of EGF4-7 and EGF11-13 and on the interdomain orientations determined using RDC data for EGF7-8, EGF8-9, and EGF10-11. The NMR data indicate that there is no fixed orientation of EGF9 relative to EGF10. Therefore, numerous relative orientations of EGF4-9 and EGF10-13 are possible. The Ca^2+^-binding EGF domains are shown in green while the other EGF domains are shown in wheat. The Ca^2+^ ions bound in EGF5, EGF7, EGF8, EGF9, EGF11, EGF12, and EGF13 are shown as red spheres.

The interdomain tilt and twist angles obtained from X-ray structures and RDC refinement allow a model for the EGF4-13 region to be constructed (Figure 4). As a result of the flexibility between EGF9 and EGF10, the model consists of two rigid segments, EGF4-9 and EGF10-13. Largely as a result of the bent interface between EGF5 and EGF6, EGF4-9 has an L shape with more extended structure in the EGF7-9 region. The model for EGF10-13 is more extended but there is a noticeable bend between EGF10 and EGF11 in contrast to the very linear EGF11-13 region.

A number of possible orientations will exist for EGF4-9 with respect to EGF10-13 as a result of the flexible EGF9-10 linker. A tight U-shaped structure has been reported for an EGF domain pair from the Merozoite surface protein 1 (Morgan et al., 1999). This structure contains a number of features not seen for Notch EGF domains including a seven-residue linker between domains and significantly longer loops between the fifth and sixth cysteines, which contain several hydrophobic residues involved in the stabilizing interdomain interface. The relatively short linker of five residues between the sixth cysteine of EGF9 and the first cysteine of EGF10 means that highly folded, U-shaped conformations are unlikely due to steric clashes.

### Implications of the EGF10-EGF13 Model for Ligand Interactions

**Figure 5:**
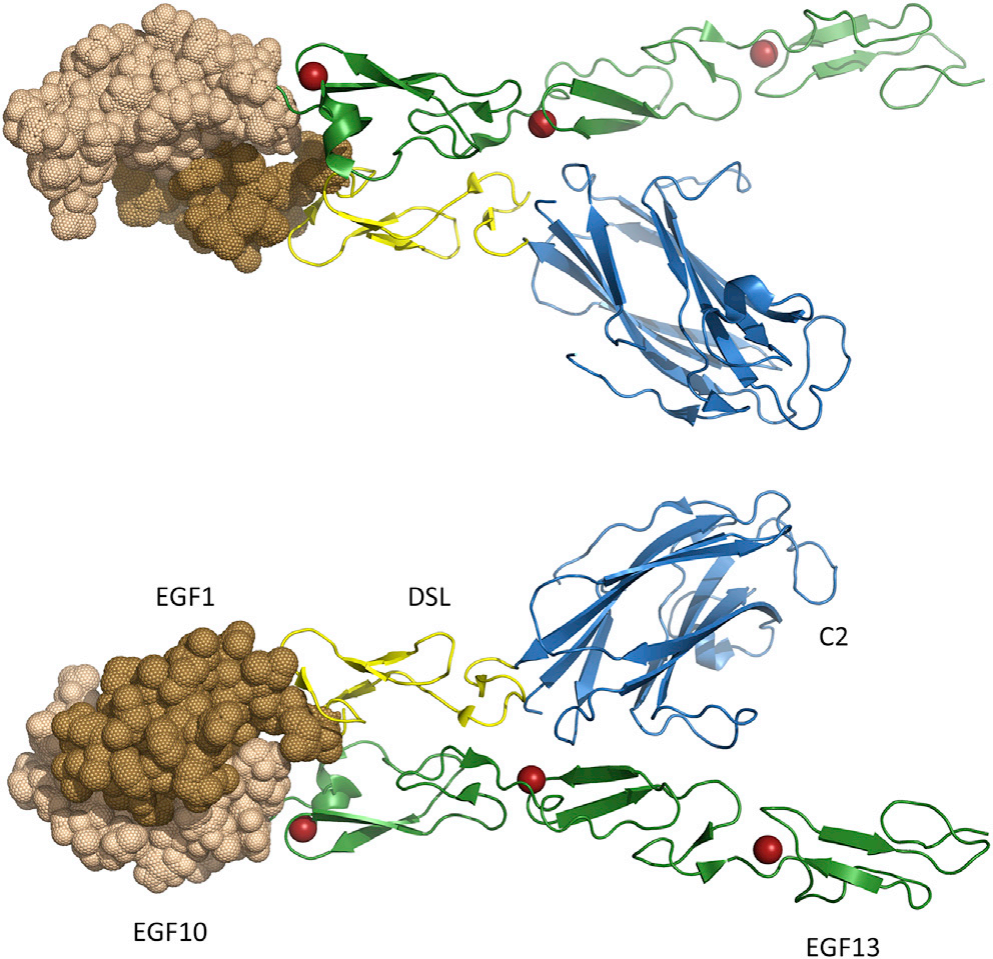
Model of Potential Interaction between EGF10 of Notch and EGF1 of Dll-4 (Top) The model of EGF10-13 has been superimposed on EGF11-13 in the Notch-Dll-4 complex (Luca et al., 2015). Notch domains EGF11-13 and the Dll-4 C2 and DSL domains are shown as a cartoon representation. EGF10 of Notch (wheat) and EGF1 of Dll-4 (light brown) are shown in a surface representation; this highlights the potential interaction between these two domains. The Ca^2+^-binding EGF domains are shown in green, the DSL domain in yellow, and the C2 domain in blue. The Ca^2+^ ions bound in EGF11, EGF12, and EGF13 are shown as red spheres. (Bottom) The model is rotated by 180° about the x axis.

The recent X-ray structure of Notch EGF11-13 in an antiparallel complex with Dll-4 shows interactions between EGF12 of Notch and the C2 domain of Dll-4 and between EGF11 of Notch and the DSL domain of Dll-4 (Luca et al., 2015). Our model for human Notch1 EGF10-13 can be used to provide further insights into Notch-ligand interactions. EGF10-13 has been superimposed onto Notch EGF11-13 in the structure of the complex solved by Luca et al. (2015) (Figure 5). This shows a potential interaction between Notch EGF10 and the EGF1 domain of Dll-4. Although there are some steric clashes between the two domains, these could be alleviated by small reorientation of the two domains. It is interesting to note that the putative interface includes residues that are not conserved between Dll and Jagged ligands, suggesting that this additional site could contribute to differences in binding.

### Comparative Binding of EGF9-13, EGF10-13, and EGF11-13 to Dll-4 and J1

**Figure 6:**
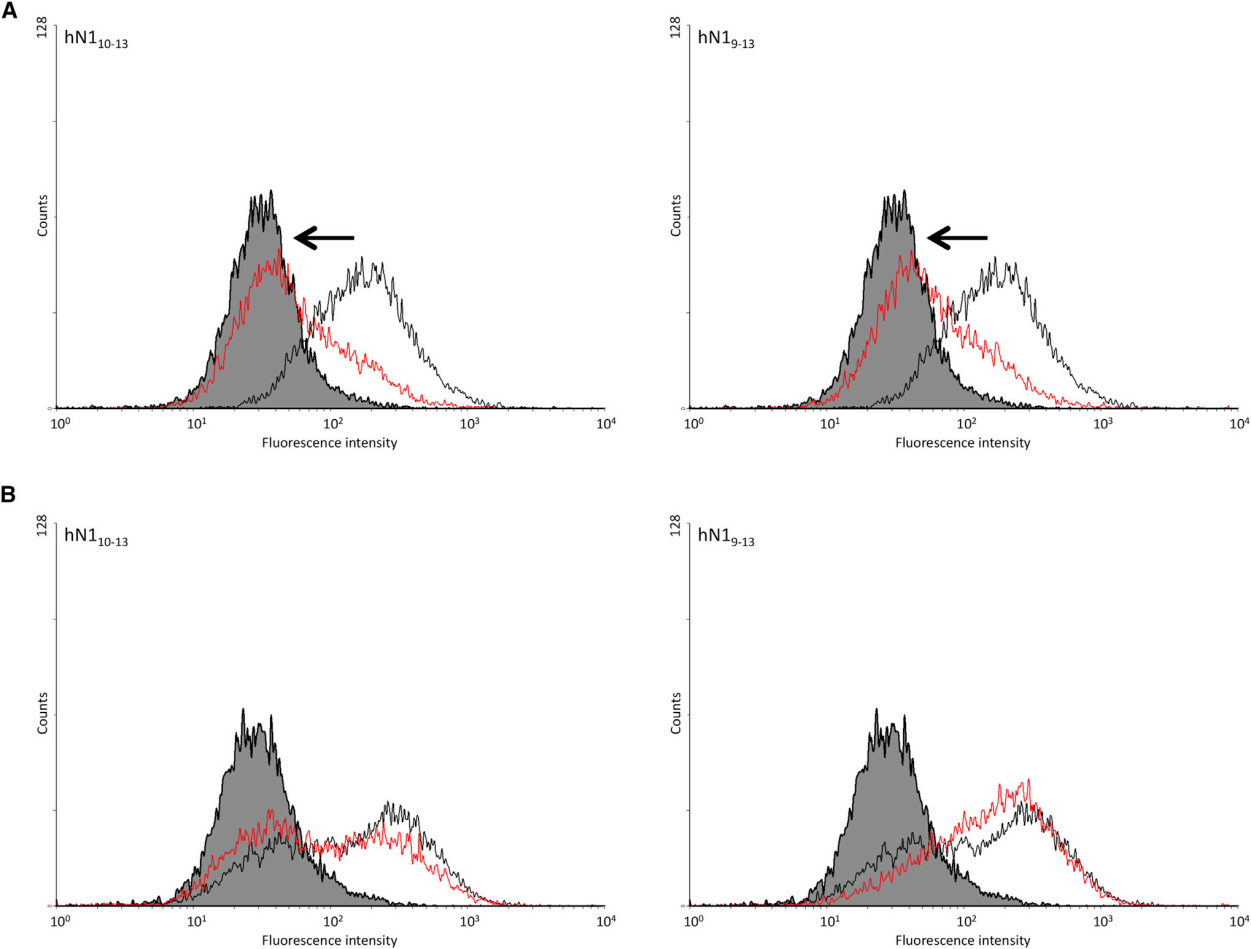
Interaction of EGF11-13, EGF10-13, and EGF9-13 Constructs of Human Notch1 with DLL-4 and J1 (A and B) Biotinylated hNotch1 EGF11-13 and (left) EGF10-13 or (right) EGF9-13 were bound to avidin-coated fluorescent beads and incubated with B16F10 cells expressing mDLL-4 (A) or mJ1 (B). The shift to the right away from the control protein (solid gray) shows binding of the EGF11-13 construct (black line) to mDLL-4 and mJ1. A reduction in binding, indicated by the black arrow, is seen for the EGF10-13 and EGF9-13 constructs (red line) to Dll-4 while no significant change is seen with mJ1.

The influence of the rigid interface between EGF10 and EGF11 on the binding to Dll-4 was probed using an established flow cytometry assay (Figure 6A) (Cordle et al., 2008b, Taylor et al., 2014). Addition of EGF10 to EGF11-13 was found to decrease binding to cells expressing full-length human Dll-4. Addition of EGF9, which has been shown in this study to have a flexible linkage to EGF10, had no further inhibitory effect on binding of EGF11-13 to Dll-4. These data suggest that the N-terminal flanking domain EGF10 modulates the binding of EGF11-13 to Dll-4 but that EGF9 does not. These experiments were repeated with Jagged1-expressing cells (Figure 6B). In contrast to Dll-4, the addition of EGF10 to EGF11-13 did not decrease the binding, indicating that the modulatory effect of EGF10 is specific to Dll-4.

## Discussion

This study has examined the effect of non-Ca^2+^-binding EGF domains on the shape of the EGF4-13 region of Notch. The majority of EGF domains in this region bind Ca^2+^, which confers the expected rigidity to the domain interface formed between the cbEGF domain and the preceding N-terminal EGF domain. Various solution NMR and X-ray structures have confirmed previously that high-affinity Ca^2+^ binding to the C-terminal EGF domain of a pair is predictive for a rod-like organization for tandem cbEGF domains and EGF-cbEGF pairs (Cordle et al., 2008a, Downing et al., 1996, Hambleton et al., 2004, Smallridge et al., 2003) and for heterologous cbEGF domain pairs (Jensen et al., 2005). In these structures, Ca^2+^ affinity is enhanced and a rigid structure is stabilized via interaction with a packing aromatic residue located between the fifth and sixth cysteine in the preceding domain (Figure S1).

The *K*_d_ values for Ca^2+^ binding to Notch EGF domains, measured by chromophoric chelation or by NMR spectroscopy, indicate moderate- to high-affinity sites (1–200 μM, *I* = 0.15, pH 7.5), which would be expected to be saturated under the physiological conditions of the extracellular milieu and insensitive to changes in Ca^2+^ flux at the cell membrane. However, the two non-Ca^2+^-binding EGF domains (EGF6 and EGF10) confer very different properties to the region. Both crystallography and NMR analysis demonstrate that the EGF5-6 interface is bent and rigid, introducing a tilt angle of ∼70°–90°, while the EGF9-10 interface is flexible. Thus, unlike the cbEGF domain, the non-Ca^2+^-binding EGF, when in a C-terminal position, can confer very different properties to a domain interface, which are not obviously predictable from sequence.

Utilizing the new structural information from this study, together with published data for the EGF11-13 ligand-binding region (Cordle et al., 2008a), it is possible to construct a new model of the EGF4-13 region. The presence of a flexible linker between EGF9-10 separates the region into two rigid halves; EGF4-9, containing the bent interface between EGF5-6, and a near-linear section comprising EGF10-13 (Figure 4). It is interesting to note that two residues, A420 and N421, in Notch1, which we showed were present in a highly flexible loop between the first and second cysteine of Notch EGF11, are observed to pack against two residues R191 and F195 in the DSL domain that are highly conserved across the two Notch ligand families and are proposed by Luca et al. (2015) “to be a conserved focal point for ligand binding.”

The rigid interface formed between EGF10-11 provides new information with which to model the receptor-ligand complex. Superposition of the EGF10-13 region on the structure of the Notch/Dll-4 complex shows that, instead of facing away from the ligand, EGF10 is in close proximity, suggesting a possible contact site between Notch EGF10 and Dll-4 EGF1 (Figure 5). Previous studies have observed that EGF1 and 2 enhance binding of the J1 N terminus, comprising the C2 and DSL domains, to Notch. This could be an indirect effect of EGF1 on DSL structural integrity and/or additional specific contacts made between ligand and receptor at the EGF1/EGF10 interface (Shimizu et al., 1999).

**Figure 7:**
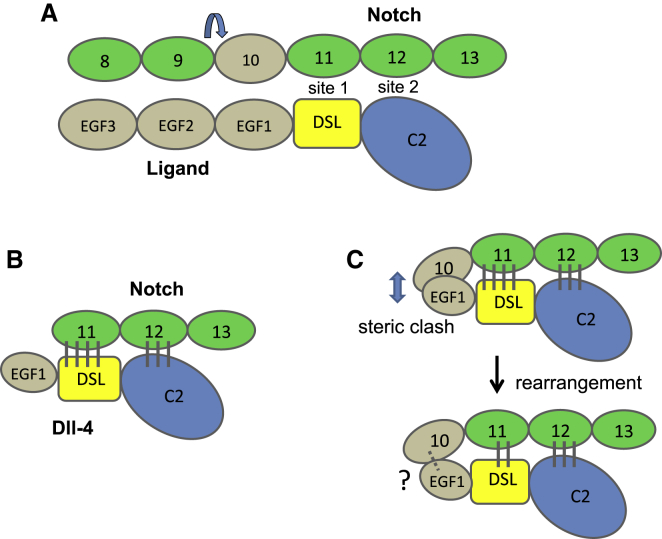
Cartoon Representation of Possible Notch/Ligand Interactions and the Effect of Addition of EGF10 on the Interaction of EGF11-13 with Ligand (A) Notch EGF11/EGF12 and Dll-4 DSL/C2 domains have been shown to interact at two sites (Luca et al., 2015). Our near-linear orientation for hN1 EGF6-9, upstream of the flexible linkage at EGF9-10 (indicated by the blue arrow above the linker), suggests that Notch may align with ligand along its longitudinal axis. The Ca^2+^-binding EGF domains are shown in green, other EGF domains in wheat, the DSL domain in yellow, and the C2 domain in blue. The Dll-1, Dll-4, J1, and J2 ligands all share the C2-DSL-EGF1-3 architecture. Dll-1 and Dll-4 have a further five EGFs while J1 and J2 have a further 13 EGFs. (B) In the X-ray structure of the Notch/Dll-4 complex, where EGF11 is in a non-native context (not bound to EGF10), EGF11 makes many more stabilizing contacts with DSL than EGF12 does with the C2 domain. The vertical lines in gray indicate stabilizing interactions between pairs of domains. (C) It is plausible that covalent linkage of EGF10 to EGF11-13 results in a steric clash between EGF10 and EGF1, and that small rearrangements that occur upon interaction with Dll-4 could disrupt some EGF11-mediated contacts within the N-terminal region of this domain. New contacts made between EGF10 and EGF1 are not sufficient to overcome the loss of EGF11-mediated contacts, since Notch EGF10-13 binds less well to Dll-4 than EGF11-13. The dashed gray line and the ? are used to indicate a possible interaction.

The five-residue flexible linker between EGF9 and 10 is likely to preclude folding back (via a U-shaped structure at EGF9-10) of the EGF4-9 region such that it would impede the core recognition region of EGF11-12 by direct interactions. Instead, the near-linear section of EGF6-9, upstream of the flexible linkage at EGF9-10, suggests that Notch may align with ligand along its longitudinal axis, and overall a number of weak interactions along the length of the molecule may contribute to the overall binding affinity of receptor to ligand (Figure 7A). EGF8 of Notch, for example, could come into close proximity with EGF3 of the ligand. This could explain the influence of mutation of a conserved residue in EGF8 (V361M), which selectively affects *Drosophila* Serrate binding (Yamamoto et al., 2012). Furthermore, post-translational O-glycosylation modifications could further stabilize this interface. Because of the flexible linker between EGF9 and EGF10, it is not possible to identify specific interaction faces from our model.

Luca et al. (2015) have postulated that, as a consequence of the antiparallel orientation of the Notch/Dll-4 complex, there may be a single Notch/ligand complex that forms at the cell surface in *cis* and in *trans*. This would necessitate a rotation, C-terminal of the core recognition region within each protein to maintain the binding interface. The identification of a flexible cbEGF/EGF linker in this study suggests that a homologous domain pair within each molecule could facilitate the necessary rotation.

Since we postulated that EGF10 was likely to make contacts with EGF1 of Dll-4 ligand, we compared the ligand binding of two fragments, EGF10-13 and EGF9-13, with that of the core recognition fragment EGF11-13. Utilizing a well-established flow cytometry assay, we demonstrated that the presence of EGF10 substantially reduced binding to Dll-4 (compared with that observed with EGF11-13). The addition of EGF9 did not further reduce binding. These data can be explained if addition of EGF10, which may have a steric clash with EGF1 of Dll-4, requires the readjustment of the positions of EGF10 and EGF1, which in turn affects the EGF11-DSL interaction site. It is notable that in the structure of the Notch/Dll-4 complex, where EGF11 is in a non-native context (not bound to EGF10), EGF11 makes many more protein:protein contacts with DSL than EGF12 does with the C2 domain. It is therefore plausible that covalent linkage of EGF10 and small rearrangements that occur on interaction with Dll-4 could disrupt some EGF11-mediated contacts within the N-terminal region of this domain (Figures 7B and 7C). If that is the case, then new contacts made between EGF10 and EGF1 are not sufficient to overcome the loss of EGF11-mediated contacts, since Notch EGF10-13 binds less well to Dll-4 than EGF11-13. The lack of any further effect of EGF9 is consistent with the flexible nature of the EGF9-10 linker.

We previously observed a similar reduction in binding to Dll-1 when comparing the binding of EGF10-14 with that of EGF11-14 and postulated a steric effect in the absence of structural data, which our current model confirms (Cordle et al., 2008b). Quantitative measurements by surface plasmon resonance showed a decreased affinity (*K*_d_ increases from 130 μM to 200 μM), indicating that not all contacts between DSL of Dll-1 and EGF11 are lost as a consequence of EGF10 addition. The importance of the EGF10-11 interface in modulating ligand binding was further shown by the introduction of a Ca^2+^-binding-site NG substitution in EGF11. This decouples the rigid interface between EGF10-11, which causes the steric clash with ligand, and restores binding. It is notable that the effect of EGF10 is ligand specific and observed only with the Delta family of ligands. This can be reconciled by the ligand-specific differences in amino acid sequences within the DSL domain, which are reflected in the substantially weaker binding of unmodified EGF11-13 to J1 compared with Dll-4 (Taylor et al., 2014), and at the proposed interface involving EGF1.

Our model for the EGF4-13 region identifies the architecture of Notch in the absence of any post-translational modification such as O-glycosylation and gives new insight into the organization of Notch/ligand complexes. Previous publications of Taylor et al. (2014) and Luca et al. (2015) have demonstrated that O-glycosylation of residues within the ligand-binding region in EGF11 and EGF12 can contribute directly to the binding interface between ligand and receptor, and many studies have indicated that O-glycosylation at other sites along Notch can influence signaling activity. Our unmodified EGF domain studies demonstrate that, in the absence of O-glycans in EGF11 and EGF12, we observe an inhibitory effect of EGF10 on Dll-4 and Dll-1 binding, but not on J1. It is therefore interesting to postulate that O-glycosyltransferase-mediated addition of O-glycans within EGF11, in addition to Fringe-mediated additions to EGF12, could be an additional mode of regulation used to modulate Notch signaling, particularly by the Delta family of ligands.

In summary, our unmodified EGF domain studies have provided new information about the shape of the Notch extracellular domain and the importance of determining the individual properties of common domain interfaces. They provide a platform to understand the basal architecture of the extracellular region of Notch, which may be further modified by O-glycosylation to fine-tune interactions with a repertoire of ligands.

## Experimental Procedures

### Protein Expression, Purification, Refolding, and Characterization

Protein expression, isotopic labeling, refolding, and purification protocols have been described previously (Muranyi et al., 2004, Weisshuhn et al., 2015a, Weisshuhn et al., 2015b, Whiteman et al., 2014). Protein fragments were expressed in *Escherichia coli* BL21 cells transformed with a pQE30 (Qiagen)-based protein expression construct and a pREP4 plasmid for control of expression via the Lac Repressor. All expression vectors contained an N-terminal His6 tag for purification and either a factor Xa (EGF4-7, EGF5-7, EGF10-13, EGF11-13) or an enterokinase (EGF8-11) recognition site for later removal of the His6 tag. The tag was not cleaved for EGF7-9 and EGF9-11; the His6 tag shows no evidence of Ca^2+^ binding. The final protein products were analyzed by SDS-PAGE (Figure S4).

### NMR Spectroscopy

All NMR experiments were carried out using spectrometers operating at ^1^H frequencies ranging from 500 to 950 MHz. The spectrometers are equipped with Oxford Instruments magnets and home-built triple-resonance pulsed-field gradient probes. Data were processed using NMRPipe (Delaglio et al., 1995) and spectra were analyzed using the CCPN software (Vranken et al., 2005).

Resonance assignments for EGF4-7, EGF8-11, and EGF11-13 have been described previously (Muranyi et al., 2004, Weisshuhn et al., 2015a, Weisshuhn et al., 2015b) (Biological Magnetic Resonance Bank accession numbers 25172, 25533, 6031). 3D ^15^N-edited total correlation spectroscopy-heteronuclear single quantum coherence (HSQC) and NOE spectroscopy (NOESY)-HSQC spectra were collected to assign the ^1^H-^15^N HSQC spectrum of EGF7-9.

Unless otherwise stated, all NMR experiments were carried out at 25°C in 5 mM Tris-HCl at pH 7.5 in 95% H_2_O/5% D_2_O. Protein samples for measurement of the {^1^H} -^15^N heteronuclear NOE or ^1^H-^15^N RDCs contained at least 25 mM CaCl_2_ to ensure all Ca^2+^-binding sites were saturated. Further information about the NOE experiments and the collection and analysis of RDC data can be found in Supplemental Experimental Procedures.

### Measurement of Ca^2+^ Dissociation Constants

For Ca^2+^ titrations monitored by NMR, protein samples were prepared in 5 mM Tris-HCl buffer (pH 7.5) made with 99.9% D_2_O containing 150 mM NaCl (to maintain approximate physiological ionic strength *I* = ∼0.15); samples were initially Ca^2+^ free and the Ca^2+^ concentration was increased by addition of CaCl_2_ aliquots up to saturating concentrations (usually >25 mM). Ca^2+^ binding was monitored using 2D ^1^H-^1^H NOESY spectra collected with a mixing time of 150 ms (Jensen et al., 2005, Smallridge et al., 1999, Suk et al., 2004).

Ca^2+^ dissociation constants for high-affinity sites were determined by competition with the chromophoric chelator 5,5′-Br_2_BAPTA (Jensen et al., 2005, Linse et al., 1991, Suk et al., 2004). Solutions of proteins in Ca^2+^-free buffer (5 mM Tris [pH 7.5], 150 mM NaCl) were titrated with Ca^2+^-stock buffer (5 mM Tris [pH 7.5], 1 mM CaCl_2_, 150 mM NaCl) in the presence of 5,5′-Br_2_BAPTA (*K*_d_ of 1.6 μM under these conditions). All titrations were performed with 20–30 μM chelator and 20–30 μM protein at room temperature (approx. 23°C) using a Shimadzu UV mini 1240 spectrophotometer. Dissociation constants were calculated by least-squares fitting to the data using in-house software (Linse et al., 1991, Stenberg et al., 1997). Each titration was repeated at least three times. Experimental data were fitted to models with one or two high-affinity Ca^2+^ binding sites, and the most suitable model was chosen using an F test. This method is suitable for defining Ca^2+^ *K*_d_ values in the range of ∼1–20 μM.

### X-Ray Crystallography

Human Notch1 EGF4-7 was crystallized by vapor diffusion from sitting drops with 25% mother liquor and protein at 14 mg/ml in 50 mM Tris (pH 7.4), 1 mM CaCl_2_. Commercially available mother liquor from Molecular Dimensions was used. The P2_1_ form crystallized in 0.1 M sodium cacodylate (pH 6.5) with 18% w/v PEG 2000 MME. The C2 form crystallized in 0.2 M imidazole malate (pH 8.5) with 7.5% w/v PEG 10,000. The Diamond facility was used for data collection (beamline I03). Both datasets were indexed and scaled using Xia2 (Winter, 2010). The structure was phased using molecular replacement of canonical EGF domains in Phaser (Mccoy et al., 2007). Structures were refined using the program Autobuster with the graphics program COOT used for manual rebuilding and inspection (Bricogne et al., 2011, Emsley and Cowtan, 2004). MolProbity was used to determine structural quality (Chen et al., 2010).

### Flow Cytometry Binding Assay

Flow cytometry was carried out as described previously (Whiteman et al., 2013). Briefly, biotinylated human Notch1 EGF11-13, EGF10-13, and EGF9-13 were coupled to avidin-coated purple fluorescent beads (Spherotech) and added to B16F10 cells expressing mDll-4 or mJagged1. Following incubation, samples were analyzed directly by flow cytometry without removal of unbound beads.

## Author Contributions

P.C.W. collected and analyzed calcium binding and NMR data. P.T. and P.W. collected and analyzed flow cytometry data. D.S. collected and refined X-ray data. S.M.L conceived and supervised the X-ray studies. P.A.H. and C.R. conceived and supervised all other aspects of the research and wrote the manuscript. All authors discussed the results and implications and commented on the manuscript at all stages.

## Figures and Tables

**Table 1:** Crystallization and Structure Determination for hN1 EGF4-7

Space Group	P2_1_	C2
Cell		
a, b, c (Å)	40.94, 86.83, 53.45	142.26, 21.15, 83.56
α, β, γ (°)	90, 107, 90	90, 116.27, 90
Wavelength (Å)	1.74626	1.74626
Resolution (Å)	43.42–2.46 (2.69–2.46)	64.73–2.92 (3.26–2.92)
*R*_merge_ (%)	3.0 (38.8)	3.1 (36.8)
I/σI	23.6 (2.3)	17.4 (2.1)
Completeness (%)	89.9 (93.8)	95.0 (98.0)
Redundancy	3.3	3
Number of reflections	39,951	15,128
*R*_work_/*R*_free_ (%)	25.9/26.3	21.4/23.7
Number of atoms		
Protein	4,154	2,099
Ligand/ion	29	8
Water	18	6
B factors		
Protein	69.2	34.4
Ligand/ion	66.0	40.5
Water	56.0	17.3
RMS deviation		
Bond length (Å)	0.035	0.01
Bond angles (°)	1.21	1.23
Residues in allowed regions of Ramachandran plot (%)	100	100
Residues in favored regions of Ramachandran plot (%)	97.3	95.4

**Table 2:** Interdomain Tilt and Twist Angles Observed in X-Ray Structures and Obtained from RDC Data

X-Ray Structures^a^			
Construct	Domain Pair	Range of Tilt Angles	Range of Twist Angles
EGF4-7	EGF4-5	33°–42°	179°–187°
EGF4-7	EGF5-6	82°–92°	112°–123°
EGF4-7	EGF6-7	25°–36°	146°–154°
EGF11-13	EGF11-12	14°–18°	119°–141°
EGF11-13	EGF12-13	10°–24°	132°–141°

RDC data^b^			
Construct	Domain Pair	Tilt Angle	Twist Angle
EGF4-7	EGF4-5	48° ± 3°	190° ± 6°
EGF4-7	EGF5-6	70° ± 2°	112° ± 7°
EGF4-7	EGF6-7	30° ± 3°	153° ± 4°
EGF7-9	EGF7-8	45° ± 2°	192° ± 17°
EGF8-11	EGF8-9	14° ± 2°	142° ± 9°
EGF8-11^c^	EGF9-10	not defined	not defined
EGF8-11	EGF10-11	33° ± 10°	172° ± 3°
EGF11-13	EGF11-12	19° ± 2°	133° ± 8°
EGF11-13	EGF12-13	16° ± 1°	149° ± 9°

a The ranges in the tilt and twist angles (Downing et al., 1996) for EGF4-7 were obtained from the two protein molecules in the P2_1_ unit cell and from the single molecule in the C2 unit cell. The ranges for EGF11-13 were obtained from several X-ray structures determined for EGF11-13 (PDB: 2VJ3, 4CUE, 4CUF, 4D0F, 4CUD, 4D0E).

b Errors in the angles are determined using an experimental error of 2 Hz for the RDCs as described in Supplemental Experimental Procedures (see also Figure S2, Tables S1 and S2).

c See also Figure S3.

## Accession Numbers

The accession numbers for the coordinates of EGF4-7 in the P2_1_ and C2 crystal forms reported in this paper are PDB: 5FMA and 5FM9.

## Supplemental Information

Document S1. Supplemental Experimental Procedures, Figures S1–S4, and Tables S1 and S2

Document S2. Article plus Supplemental Information
