# Supplementary material for: Non-Linear and Flexible Regions of the Human Notch1 Extracellular Domain Revealed by High-Resolution Structural Studies
Source: Structure. 2016 Apr 5;24(4):555–66. doi: 10.1016/j.str.2016.02.010 (PMC4826273; doi:10.1016/j.str.2016.02.010)
Supplement: Document S1. Supplemental Experimental Procedures, Figures S1–S4, and Tables S1 and S2 [file mmc1.pdf]

Structure, Volume *24*

Supplemental Information

Non-Linear and Flexible Regions of the Human Notch1 Extracellular Domain Revealed by High-Resolution Structural Studies

Philip C. Weisshuhn, Devon Sheppard, Paul Taylor, Pat Whiteman, Susan M. Lea, Penny A. Handford, and Christina Redfield

(A)

**hN-1 4**	-	ADP	C	ASNP	C	ANGGQ	C	LPFEASYI	C	H	C	PPSFHGPT	C RQ
**hN-1 5**	calcium	DVNE	C	GQKPGL	C	RHGGT	C	HNEVGSYR	C	V	C	RATHTGPN	C ER
**hN-1 6**	-	PYVP	C	SPSP	C	QNGGT	C	RPTGDVTHE	C	A	C	LPGFTGQN	C EE
**hN-1 7**	calcium	NIDD	C	PGNN	C	KNGGA	C	VDGVNTYN	C	R	C	PPEWTGQY	C TE
**hN-1 8**	calcium	DVDE	C	QLMPNA	C	QNGGT	C	HNTHGGYN	C	V	C	VNGWTGED	C SE
**hN-1 9**	calcium	NIDD	C	ASAA	C	FHGAT	C	HDRVASFY	C	E	C	PHGRTGLL	C HL
**hN-1 10**	-	NDA	C	ISNP	C	NEGSN	C	DTNPVNGKAI	C	T	C	PSGYTGPA	C SQ
**hN-1 11**	calcium	DVDE	C	SLGANP	C	EHAGK	C	INTLGSFE	C	Q	C	LQGYTGPR	C EI
**hN-1 12**	calcium	DVNE	C	VSNP	C	QNDAT	C	LDQIGEFQ	C	I	C	MPGYEGVH	C EV
**hN-1 13**	calcium	NTDE	C	ASSP	C	LHNGR	C	LDKINEFQ	C	E	C	PTGFTGHL	C QY

(B)

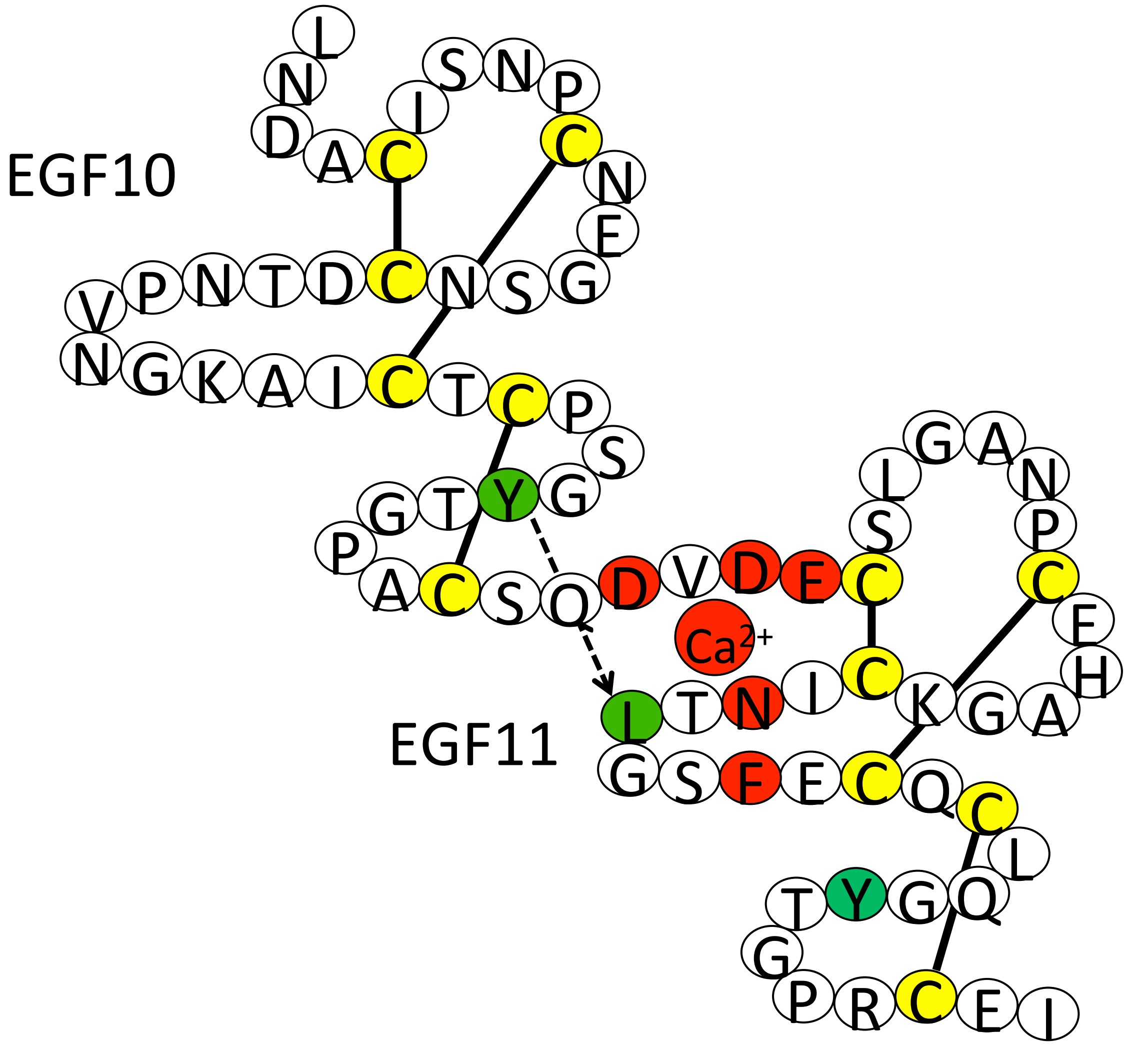

Figure S1

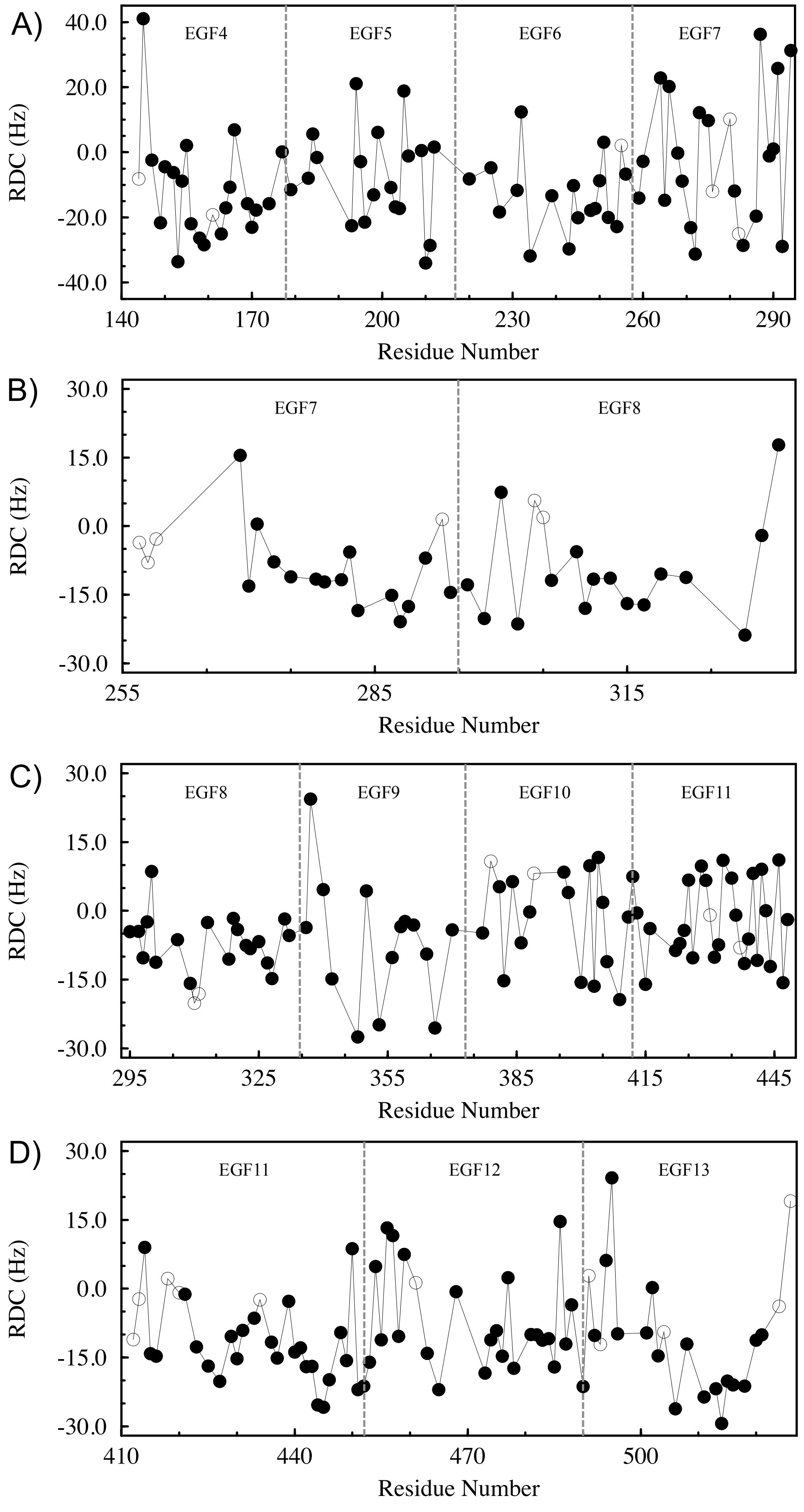
A)
B)
C)
D)
EGF4
EGF5
EGF6
EGF7
EGF8
EGF9
EGF10
EGF11
EGF12
EGF13
RDC (Hz)
Residue Number
40.0
20.0
0.0
-20.0
-40.0
30.0
15.0
-15.0
-30.0
140
170
200
230
260
290
255
285
315
295
325
355
385
415
445
410
440
470
500

Figure S2

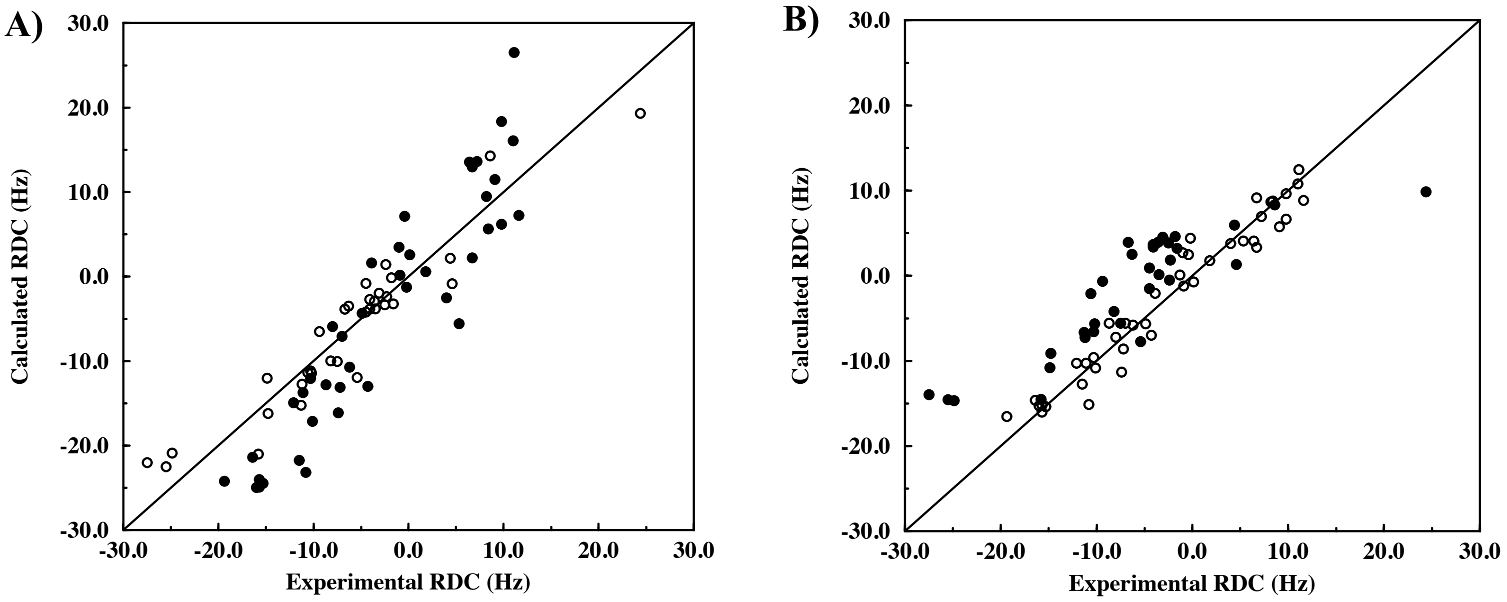
A)
B)
Calculated RDC (Hz)
Experimental RDC (Hz)
30.0
20.0
10.0
0.0
-10.0
-20.0
-30.0
-30.0
-20.0
-10.0
0.0
10.0
20.0
30.0

Figure S3

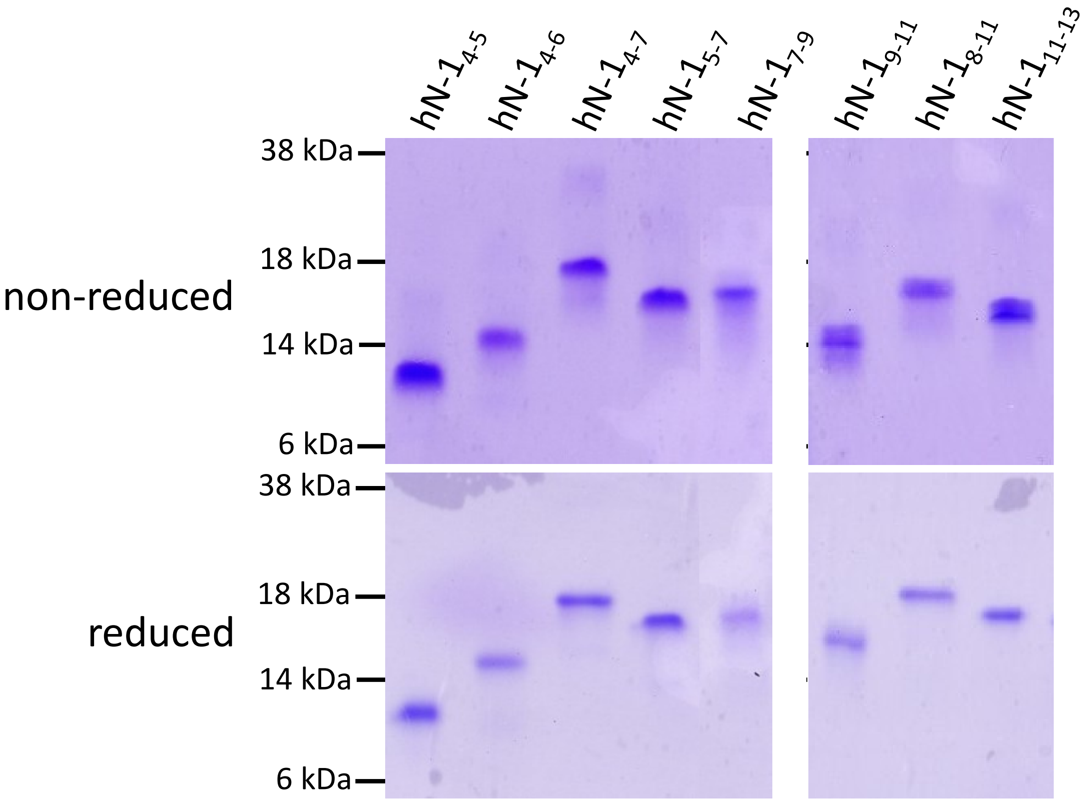

hN-1$_{4-5}$
hN-1$_{4-6}$
hN-1$_{4-7}$
hN-1$_{5-7}$
hN-1$_{7-9}$
hN-1$_{9-11}$
hN-1$_{8-11}$
hN-1$_{11-13}$
non-reduced
reduced
38 kDa
18 kDa
14 kDa
6 kDa
38 kDa
18 kDa
14 kDa
6 kDa

Figure S4

Supplemental Figure Legends

Figure S1. Related to Figure 1. Sequence alignment of EGF domains 4-13 in hN-1 showing consensus calcium-binding residues and schematic representation of an EGF/calcium-binding EGF pair
(A) Alignment was performed on conserved cysteines (yellow). Most EGF domains are Ca^{2+} binding. The 5 solid red arrows indicate the positions of the conserved residues of the D/N-x-D/N-E/Q-x_m-D/N*-x_n-Y/F calcium-binding motif. The green arrows indicate the position of the conserved hydrophobic residue in the β-hairpin and the aromatic residue involved in a packing interaction with the following domain.
(B) Schematic representation of the EGF10-EGF11 pair. Each EGF domain contains six highly-conserved cysteine residues (shown in yellow) paired in a 1-3, 2-4, 5-6 arrangement to stabilize domain structure. There are typically 6 residues between the 6th cysteine of an EGF domain and the 1st cysteine of the following domain. In EGF11, the residues of the consensus calcium-binding sequence, D/N-x-D/N-E/Q-x_m-D/N*-x_n-Y/F (where * indicates possible β-hydroxylation and m/n are variable), and the calcium ion are indicated in red. The residues normally involved in interdomain packing, the aromatic residue four positions after the 5th cysteine in the N-terminal domain and the hydrophobic residue in the β-hairpin of the C-terminal domain, are indicated in green. In EGF10/EGF11 this interaction involves Y404 and L433 and is indicated with an arrow. Y444 is involved in an interdomain packing interaction with EGF12.

Figure S2. Related to Figure 2 and Table 2. Residual dipolar coupling (RDC) data for EGF4-7, EGF7-8, EGF8-11 and EGF11-13
(A) RDCs measured for a total of 83 residues in EGF4-7 in 2.5% C12E6/*n*-hexanol are plotted as a function of sequence. Even in this low concentration of the alignment medium, large RDC values ranging from -40 to +40 Hz were observed.
(B) The EGF7-9 construct showed broadened peaks for EGF9 but sharp peaks for EGF7 and EGF8. RDCs measured for a total of 37 residues in EGF7-8 in 2.5% C12E6/*n*-hexanol are plotted as a function of sequence.
(C) RDCs measured for a total of 81 residues in EGF8-11 in 2.7% C12E6/*n*-hexanol are plotted as a function of sequence.
(D) RDCs measured for a total of 79 residues in EGF11-13 in 2% C12E6/*n*-hexanol are plotted as a function of sequence. Even in this low concentration of the alignment medium, large RDC values ranging from -30 to +30 Hz were observed; this is consistent with an elongated structure for EGF11-13. RDC values excluded from the fitting procedures are shown as open circles. The domain boundary is indicated by a dashed vertical line.

Figure S3. Related to Table 2. RDC data do not support a defined interdomain interface for EGF9-EGF10
(A) The D_a and R values (14.9 and 0.48) obtained from fits of the EGF8-9 RDC data are used to obtain a best fit between experimental and calculated RDCs for EGF10-11 (only the angles θ, ϕ, ψ are optimised). The RDC data for EGF8-9 are represented by open circles and the RDC data for EGF10-11 by filled circles. Q values of 0.27 and 0.68 are obtained for the EGF8-9 and EGF10-11 RDCs, respectively.
(B) The D_a and R values (-8.5 and 0.38) obtained from fits of the EGF10-11 RDC data are used to obtain a best fit between experimental and calculated RDCs for EGF8-9 (only the angles θ, ϕ, ψ are optimised). The RDC data for EGF10-11 are represented by open circles and the RDC data for EGF8-9 by filled circles. Q values of 0.22 and 0.59 are obtained for the EGF10-11 and EGF8-9 RDCs, respectively.

Figure S4. Related to Figure 1. SDS-PAGE of constructs used in this study
Top panels show non-reduced material, whereas the bottom panel shows reduced material. All constructs show a good degree of purity under both reducing and non-reducing conditions. The SeeBlue Plus2 Pre-Stained Standard was used as the protein standard. Electrospray ionisation mass spectrometry was performed to check the correct mass of each construct. The following were the principle human Notch1 constructs used in this study: EGF4-7 (residues Q140 to E294), EGF5-7 (residues D178 to E294), EGF7-9 (residues N257 to L372), EGF8-11 (residues D295 to I451), EGF9-11 (residues N335 to I451), EGF10-13 (N373 to P517), EGF11-13 (residues D412 to P517).

Table S1. Related to Figure 2 and Table 2. Fits of RDC data for individual EGF domains in the constructs studied

Construct	Domain	Number of measured RDCs	Number of RDCs used in fits	Q value	D_a	R
EGF4-7[a]	EGF4	22	20	0.14	20.6 ± 0.6	0.33 ± 0.03
EGF4-7[a]	EGF5	19	19	0.21	21.0 ± 0.8	0.35 ± 0.04
EGF4-7[a]	EGF6	19	17	0.20	20.8 ± 0.6	0.26 ± 0.03
EGF4-7[a]	EGF7	23	20	0.14	19.4 ± 0.6	0.42 ± 0.04
EGF7-9[b]	EGF7	19	16	0.18	18.3 ± 0.8	0.27 ± 0.03
EGF7-9[b]	EGF8	18	14	0.14	19.1 ± 1.0	0.21 ± 0.05
EGF8-11[c]	EGF8	21	19	0.24	11.7 ± 0.7	0.41 ± 0.04
EGF8-11[c]	EGF9	14	14	0.12	17.9 ± 0.8	0.50 ± 0.04
EGF8-11[c]	EGF10	18	16	0.17	-9.2 ± 0.5	0.52 ± 0.13
EGF8-11[c]	EGF11	28	26	0.23	-8.0 ± 0.4	0.37 ± 0.09
EGF11-13[d]	EGF11	30	25	0.13	18.7 ± 0.5	0.26 ± 0.02
EGF11-13[d]	EGF12	26	25	0.24	17.6 ± 0.6	0.27 ± 0.03
EGF11-13[d]	EGF13	23	18	0.16	21.0 ± 0.7	0.26 ± 0.03

[a]For EGF4, EGF5, EGF6 and EGF7 subsets of the measured RDCs were found to agree well with predictions from the X-ray structures of individual domains. The four domains give D_a and R values that are all similar suggesting that the molecule is rigid and tumbles in solution as a rigid object.

[b]An X-ray structure for EGF7-9 is not available. EGF7 is part of the EGF4-7 construct for which an X-ray structure exists. The coordinates of EGF7 from EGF4-7 were used to assess if the conformation of this calcium-binding domain was altered in a non-native context lacking an interdomain packing interaction in the absence of EGF6. Good agreement between experimental and calculated RDCs is obtained if residues at the N- and C-termini of EGF7, which have a different environment in EGF7-9 than in EGF4-7, are excluded. This demonstrates that the core structure of EGF7 is not altered when it is preceded or followed by EGF6 or EGF8. Alignment of the sequences of calcium-binding EGF domains for which X-ray coordinates exist with the sequence of EGF8 showed the best match for EGF11, in terms of loop length between the 1st and 2nd cysteines and between the 3rd and 4th cysteines (Figure S1). EGF11 is found to be a good model for EGF8. The EGF7 and EGF8 domains give D_a and R values that are similar suggesting that the molecule is rigid and tumbles in solution as a rigid object.

[c]An X-ray structure for EGF8-11 is not available. The EGF8-9 pair was modelled using EGF11-12; EGF8 and EGF11 both have 6 residues in the loop between the 1st and 2nd cysteines while EGF9 and EGF12 have 4 residues in this loop (Figure S1). Both EGF8 and 9, like EGF11 and 12, are calcium-binding domains. The RDCs for EGF11 measured in the EGF8-11 construct were fitted to the X-ray structure of EGF11 in the EGF11-13 construct; residue 412, at the N-terminus, and 450, at the C-terminus, were excluded because they are found in a different context in EGF8-11 and EGF11-13. This shows that the structure of EGF11 does not change significantly when it is preceded by EGF10. On the basis of sequence alignments, EGF10 was modelled using EGF22 from the EGF21-23 X-ray structure; EGF10 and EGF22 are both non-calcium binding domains and have 4 residues in the loop between the 1st and 2nd cysteines. EGF22 is found to be a good model for EGF10.

[d]For EGF11, EGF12 and EGF13 subsets of the measured RDCs were found to agree well with predictions from the X-ray structures of individual domains. In EGF11, 4 of the 5 residues giving poor agreement correspond to residues at the N-terminus (D412, V413) or in the loop between the 1st and 2nd cysteines (L418, A420), which was shown to be mobile in the $\{^{1}H\}$ -^{15}N heteronuclear NOE experiment. The three domains give D_a and R values that are all similar suggesting that the molecule is rigid and tumbles in solution as a rigid object.

Table S2. Related to Figure 2 and Table 2. Fits of RDC data for EGF domain pairs in the constructs studied

Construct	Domain pair	Number of RDCs used in fits	Q value (domain orientation optimized)[a]	D_a	R
EGF4-7[b]	EGF4-5	39	0.17	20.7 ± 0.5	0.34 ± 0.02
EGF4-7[b]	EGF5-6	36	0.21	20.9 ± 0.5	0.29 ± 0.02
EGF4-7[b]	EGF6-7	37	0.17	20.3 ± 0.4	0.32 ± 0.02
EGF7-9[c]	EGF7-8	30	0.17	18.5 ± 0.6	0.26 ± 0.02
EGF8-11[d]	EGF8-9	33	0.27<br>(0.20)[e]	14.9 ± 0.5<br>(18.0 ± 0.5)	0.48 ± 0.03<br>(0.45 ± 0.02)
EGF8-11[f]	EGF10-11	42	0.22	-8.5 ± 0.3	0.38 ± 0.08
EGF11-13[g]	EGF11-12	50	0.19	18.2 ± 0.4	0.27 ± 0.02
EGF11-13[g]	EGF12-13	43	0.21	19.1 ± 0.4	0.29 ± 0.02

[a]A subset of RDCs from a domain pair was fitted simultaneously to the X-ray structures of two EGF domains. Global values of D_a and R, defining the axial component of the alignment tensor and the rhombicity, were used for all residues in the pair but the relative orientation of the domains (as defined by the Euler angles θ, ϕ, ψ) was allowed to vary to optimise the fit (by minimising the Q value). The influence of experimental error of 2 Hz in the measured RDCs on the fitted parameters D_a, R, θ, ϕ, ψ and the interdomain tilt and twist angles was assessed as described in the Supplemental Experimental Procedures.

[b]Fitting of the RDC data for the EGF4-5, EGF5-6 and EGF6-7 domain pairs was carried out using the X-ray structure of EGF4-7. The interdomain tilt and twist angles from the RDC data are found to be 48° ± 3° and 190° ± 6° for EGF4-5, 70° ± 2° and 112° ± 7° for EGF5-6 and 30° ± 3° and 153° ± 4° for EGF6-7. For all domain pairs, the twist angles determined from the RDC data fall within the range of values observed in the X-ray structures (Table 2). This is also true for the tilt angle determined for EGF6-7. For EGF4-5, the tilt angle is slightly higher than the range of values observed in X-ray structures. For EGF5-6, the tilt angle observed in solution is ~10° smaller than the smallest value observed in the X-ray structures indicating that, in solution, the EGF5-6 interface is less bent.

[c]Fitting of the RDC data for the EGF7-8 domain pair was carried out using the X-ray structures of EGF7 (from EGF4-7) and EGF11 (from EGF11-13). The EGF7-8 tilt and twist angles determined from the RDC data are 45° ± 2° and 192° ± 17.

[d]Fitting of the RDC data for the EGF8-9 domain pair was carried out using the X-ray structure of EGF11-12 (from EGF11-13). Tilt and twist angles of 14° ± 2° and 142° ± 9° are determined. The EGF11-12 pair has a tilt angle of 14° and a twist angle of and 120° in the 2VJ3 X-ray structure so it is the twist of the two domains that is altered in EGF8-9.

[e]It is noticeable that EGF8 has a significantly lower D_a value than EGF9 (Table S1). This may result from some averaging of EGF8, the N-terminal domain, with respect to the rest of the construct. If the RDC values of EGF8 are scaled up by ~1.5 relative to those of EGF9 then the Q value for the pair decreases from 0.27 to 0.20. Interestingly, the relative orientation of the two domains is not changed significantly with tilt and twist angles of 13° ± 2° and 141° ± 8°.

[f]Fitting of the RDC data for the EGF10-11 domain pair was carried out using the X-ray structures of EGF11 (from EGF11-13) and EGF22 (from EGF21-23). The EGF10-11 tilt and twist angles from the RDC data are found to be 33° ± 10° and 172° ± 3°. The D_a values obtained from the fits of the RDCs for EGF8-9 and EGF10-11 are significantly different in both their magnitude and sign (D_a = 14.9 ± 0.5 and R = 0.48 ± 0.03 for EGF8-9 and D_a = -8.5 ± 0.3 and R = 0.38 ± 0.08 for EGF10-11). Attempts to simultaneously fit the RDC data for the four domains to a single value of D_a and R gives a significantly higher Q value than the individual fits of EGF8-9 and EGF10-11. Fitting of the RDC data for EGF8-9 using the D_a and R values obtained for EGF10-11 results

in a large Q value of 0.59. Similarly, fitting of the EGF10-11 RDCs using the D_a and R values for EGF8-9 results in a large Q value of 0.68 (Figure S3). This suggests that the two pairs of domains in the EGF8-11 align independently in solution.

[g]Fitting of the RDC data for the EGF11-12 and EGF12-13 domain pairs was carried out using the X-ray structure of EGF11-13. The interdomain tilt and twist angles between EGF11 and EGF12 were found to be 19º ± 2º and 133º ± 8º. The interdomain tilt and twist angles between EGF12 and EGF13 were found to be 16º ± 1º and 149º ± 9º. Within experimental error, the interdomain tilt and twist angles determined from the RDC data for the EGF11-13 construct fall within the range of values observed in the ensemble of X-ray structures (Table 2).

SUPPLEMENTAL EXPERIMENTAL PROCEDURES

NMR spectroscopy

$\{^1H\}$ -^{15}N heteronuclear NOE experiments were carried out on ^{15}N-labelled protein samples in order to examine the sub-nanosecond dynamics of specific amides (Kay et al., 1989). Spectra with and without ^1H saturation were collected as interleaved experiments. The $\{^1H\}$ -^{15}N NOE was calculated as the ratio of the peak intensities in the spectra recorded with and without ^1H saturation. Peak heights were determined using in-house peak-picking software. Uncertainties in the NOE ratios were estimated from 500 Monte Carlo simulations using baseline noise as a measure of the error in the peak heights. Data for the EGF4-7 and EGF8-11 constructs were collected at a ^1H frequency of 750 MHz. Data for the EGF7-9 and EGF11-13 constructs were collected at 950 and 600 MHz, respectively. ^1H saturation was applied for 4 s at 600 and 750 MHz and for 4.5 s at 950 MHz. The data sets were acquired with 1K complex points in F_2 and 128 complex t_1 increments. 96, 128, 96 and 80 scans were collected for EGF4-7, EGF7-9, EGF8-11 and EGF11-13, respectively.

Residual dipolar couplings (RDCs) were collected for the EGF4-7, EGF7-9, EGF8-11 and EGF11-13 constructs using liquid crystalline media containing n-alkyl-poly(ethylene glycols) (PEG) and *n*-alkyl alcohols as described previously (Ruckert and Otting, 2000). Isotropic spectra were first collected for protein solutions in 90% H_2O/10% D_2O, >25 mM calcium, at pH 7.5 using the interleaved IPAP experiment (Ottiger et al., 1998). A 15% stock C12E6/*n*-hexanol solution was prepared by adding 18μl of *n*-hexanol to 500μl of a 15% C12E6 solution in 90% H_2O/10% D_2O, >25 mM calcium at pH 7.5. Aligned protein samples were prepared by adding an appropriate aliquot of the 15% C12E6/hexanol stock to the protein solution used for the isotropic measurement. The concentration of C12E6/hexanol used for the EGF4-7, EGF7-9, EGF8-11 and EGF11-13 samples was 2.5%, 2.5%, 2.7% and 2%, respectively. IPAP experiments were performed at a ^1H frequency of 600 MHz at 25 ^oC using 128 and 1024 complex points in F_1 (^{15}N) and F_2 (^1H), respectively. Residual dipolar couplings were measured as the difference between the splitting observed in the isotropic and aligned data sets.

Analysis of RDC data to define interdomain orientation

RDC data were used to define the interdomain orientation in solution of pairs of EGF domains. Domain pairs extracted from the X-ray coordinates of human Notch1 EGF4-7, EGF11-13 and EGF21-23 were used in the fitting procedure. The choice of an appropriate model for fitting to the experimental RDCs was based on a sequence alignment of human Notch1 EGF domains and the number of residues between pairs of adjacent cysteines in the sequences of the domains. The number of residues between the 1st and 2nd cysteine and between the 3rd and 4th cysteine varies in the EGF domains studied here. In contrast, the number of residues between cysteines 2-3, 4-5 and 5-6 is constant (Figure S1).

Relative domain orientation was determined as follows. First, the RDC values for the individual domains were fitted to X-ray coordinates of individual EGF domains, using an in-house program, to identify a subset of residues that give a good fit (Table S1). The overall fit between experimental and calculated RDC values was assessed using the Q value, defined as:

$Q = [\sum_{i=1,\ldots,N} (RDC^{expt} - RDC^{calc})^2 / N]^{½} / RDC_{rms}$ (Cornilescu et al., 1998).

Residues with very poor fits generally were located in loop regions where local structure is less conserved between EGF domains; these were excluded from further fits. In addition, residues identified as flexible on the basis of the $\{^1H\}$ -^{15}N heteronuclear NOE experiment were also excluded.

In the second phase, the subsets of RDCs from a pair of domains were fitted simultaneously to the X-ray structures of two EGF domains. Global values of D_a and R, defining the axial component of the alignment tensor and the rhombicity, were used for all residues in the pair but the relative orientation of the domains (as defined by the angles θ, ϕ, ψ) was allowed to vary to optimise the fit (by minimising the Q value) (Table S2).

The influence of experimental error in the measured RDCs on the fitted parameters D_a, R, θ, ϕ, ψ was assessed as follows. Fits of the experimental RDCs to the X-ray structures were repeated 500 times in Monte Carlo simulations in which an experimental error of 2 Hz on the RDCs was assumed. The observed variation in the two sets of angles (θ, ϕ, ψ) which define the relative orientation of the two alignment tensors, was propagated through to determine the variation in

interdomain tilt and twist angles (Table 2). These angles were calculated using the program mod2 using the positions of residues within the major β-hairpin as the reference point (Bork et al., 1996; Downing et al., 1996). The beginning and end of each EGF domain were defined as the residue 4 before the 1st cysteine and the residue 2 after the 6th cysteine, respectively.

A single set of RDC values will not predict a unique interdomain orientation. When an X-ray structure was used as the starting point for the RDC fitting, the optimised structure with the interdomain orientation closest to the starting X-ray structure was selected. In other cases, generally two of the possible interdomain orientations were eliminated due to steric clashes resulting from the short interdomain linker. Of the two remaining interdomain orientations, the one which gave a structure with the expected packing interaction between the aromatic residue four after the 5th cysteine in the N-terminal domain and the residues in the major b-term of the C-terminal domain was selected.

Supplemental References

Bork, P., Downing, A.K., Kieffer, B., and Campbell, I.D. (1996). Structure and distribution of modules in extracellular proteins. Q Rev Biophys *29*, 119-167.

Cornilescu, G., Marquardt, J.L., Ottiger, M., and Bax, A. (1998). Validation of protein structure from anisotropic carbonyl chemical shifts in a dilute liquid crystalline phase. J Am Chem Soc *120*, 6836-6837.

Kay, L.E., Torchia, D.A., and Bax, A. (1989). Backbone Dynamics of Proteins as Studied by N-15 Inverse Detected Heteronuclear Nmr-Spectroscopy - Application to Staphylococcal Nuclease. Biochemistry-Us *28*, 8972-8979.

Ottiger, M., Delaglio, F., and Bax, A. (1998). Measurement of J and dipolar couplings from simplified two-dimensional NMR spectra. J Magn Reson *131*, 373-378.
